# Racial/Ethnic Disparities and Survival Characteristics in Non-Pancreatic Gastrointestinal Tract Neuroendocrine Tumors

**DOI:** 10.3390/cancers12102990

**Published:** 2020-10-15

**Authors:** Suleyman Yasin Goksu, Muhammet Ozer, Muhammad S. Beg, Nina Niu Sanford, Chul Ahn, Benjamin D. Fangman, Busra B. Goksu, Udit Verma, Aravind Sanjeevaiah, David Hsiehchen, Amy L. Jones, Radhika Kainthla, Syed M. Kazmi

**Affiliations:** 1 Department of Internal Medicine, UT Southwestern Medical Center, Dallas, TX 75390, USA; suleyman.goksu@utsouthwestern.edu (S.Y.G.); muhammad.beg@utsouthwestern.edu (M.S.B.); benjamin.fangman@phhs.org (B.D.F.); busnurb.goksu@gmail.com (B.B.G.); udit.verma@utsouthwestern.edu (U.V.); aravind.sanjeevaiah@utsouthwestern.edu (A.S.); david.hsieh@utsouthwestern.edu (D.H.); amy.jones@utsouthwestern.edu (A.L.J.); radhika.kainthla@utsouthwestern.edu (R.K.); 2 Division of Hematology and Oncology, UT Southwestern Medical Center, Dallas, TX 75390, USA; mozer@capitalhealth.org; 3 Department of Internal Medicine, Capital Health Regional Medical Center, Trenton, NJ 08638, USA; 4 Department of Radiation Oncology, UT Southwestern Medical Center, Dallas, TX 75390, USA; nina.sanford@utsouthwestern.edu; 5 Department of Population and Data Sciences, UT Southwestern Medical Center, Dallas, TX 75390, USA; chul.ahn@utsouthwestern.edu

**Keywords:** neuroendocrine tumors, gastrointestinal tract, survival analysis, database, race factors

## Abstract

**Simple Summary:**

The impact of race and ethnicity on survival characteristics in non-pancreatic gastrointestinal tract neuroendocrine tumors is understudied. We evaluated the survival outcomes and racial/ethnic disparities in the gastrointestinal tract neuroendocrine tumors, including the esophagus, stomach, small intestine, colon, rectum, and appendix. Survival trends were determined among three groups: Hispanic, non-Hispanic White, and non-Hispanic Black. We analyzed a large national database and found that race/ethnicity is an independent prognostic factor in patients with gastrointestinal neuroendocrine tumors. Hispanic patients had better overall survival than non-Hispanic White patients, whereas non-Hispanic Black patients had favorable cause-specific survival compared to non-Hispanic White patients. This survival disparity can be attributed to differences in the site of origin, age, and stage at presentation between various race/ethnicity. Understanding these differences between race and ethnicity is needed to reduce disparities in cancer outcomes.

**Abstract:**

*Background*: We studied the effect of race and ethnicity on disease characteristics and survival in gastrointestinal neuroendocrine tumors. *Methods*: The Surveillance, Epidemiology, and End Results database was used to select patients with non-pancreatic gastrointestinal neuroendocrine tumors diagnosed between 2004 and 2015. Trends in survival were evaluated among three groups: Hispanic, non-Hispanic White, and non-Hispanic Black. Kaplan–Meier and Cox regression methods were performed to calculate overall survival and cause-specific survival after adjusting for patient and tumor characteristics. *Results:* A total of 26,399 patients were included in the study: 65.1% were non-Hispanic White, 19.9% were non-Hispanic Black, and 15% were Hispanic. Non-Hispanic White patients were more likely to be male (50.0%, *p* < 0.001), older than 60 years (48.0%, *p* < 0.001), and present with metastatic disease (17.7%, *p* < 0.001). Non-Hispanic White patients had small intestine neuroendocrine tumors, while Hispanic and non-Hispanic Black patients had rectum neuroendocrine tumors as the most common primary site. Hispanic patients had better overall survival, while non-Hispanic Black patients had better cause-specific survival versus non-Hispanic White patients. This finding was confirmed on multivariable analysis where Hispanic patients had improved overall survival compared to non-Hispanic White patients (Hazard ratio (HR): 0.89 (0.81–0.97)), whereas non-Hispanic Black patients had better cause-specific survival compared to non-Hispanic White patients (HR: 0.89 (0.80–0.98)). *Conclusions*: Race/ethnicity is an independent prognostic factor in patients with gastrointestinal neuroendocrine tumors.

## 1. Introduction

Neuroendocrine tumors are rare and heterogeneous type of tumors caused by the malignant transformation of the neuroendocrine cells of many different organs [1]. Gastrointestinal neuroendocrine tumors constitute approximately 60% of all neuroendocrine tumors, with the small intestine being the most frequent site of origin, followed by the rectum, colon, and stomach [2,3]. Each anatomical location has distinct clinical, pathological characteristics, treatment response, and prognosis [4,5].

The worldwide burden of neuroendocrine tumors, and more specifically gastrointestinal tract neuroendocrine tumors, has increased over the last several decades due to increased use of diagnostic and screening studies (endoscopy, computer tomography, etc.), immunohistochemical sensitivity, and awareness in physicians [6,7]. According to Surveillance, Epidemiology, and End Results (SEER), the incidence rate of neuroendocrine tumors has increased from 1.09/100,000 to 6.9/100,000 [5,6,8,9,10], and the small intestine and rectal neuroendocrine tumors incidence rate has risen more than other gastrointestinal tract neuroendocrine tumors [11]. Besides the increased usage of diagnostic tests, other risk factors associated with the development of gastrointestinal tract neuroendocrine tumors include diet, environmental exposures, and use of profound acid suppression by proton pump inhibitors [12]. Although the annual incidence of gastrointestinal tract neuroendocrine tumors has been increasing worldwide, prognostic factors related to survival outcomes are not well described. In contrast, racial disparities have been associated with variable survival outcomes in solid malignancies, including pancreatic neuroendocrine tumor, prostate, colorectal, and female breast neoplasms [13,14,15,16]. To date, the impact of race and ethnicity on non-pancreatic gastrointestinal tract neuroendocrine tumors has not been well studied. The primary objective of this study was to determine survival outcomes and racial/ethnic disparities in the gastrointestinal tract neuroendocrine tumors, including the esophagus, stomach, small intestine, colon, rectum, and appendix using the SEER database. The effect of race on pancreatic neuroendocrine tumors has been reported before.

## 2. Results

### 2.1. Baseline Characteristics, Demographics, and Tumor Characteristics

**Figure 1:**
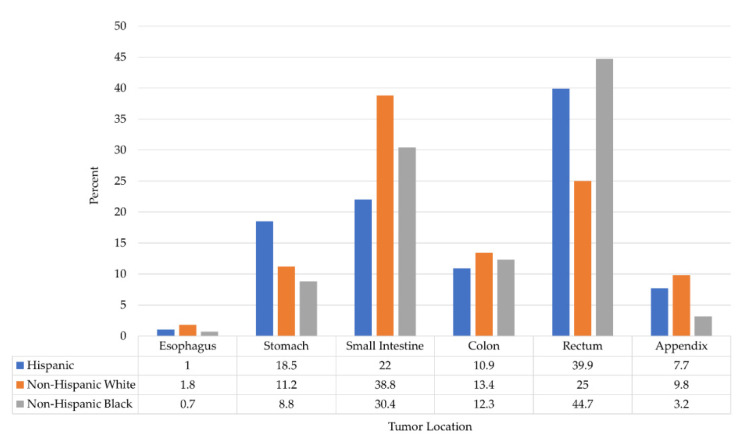
Distribution of gastrointestinal neuroendocrine tumors between race and ethnicity.

Of the 26,399 patients with gastrointestinal tract neuroendocrine tumors, 17,183 (65.1%) were non-Hispanic White, 5256 (19.9%) were non-Hispanic Black, and 3960 (15%) were Hispanic. Hispanic patients were more likely to be younger (age less than 40 years) compared to others (*p* < 0.001). Non-Hispanic White patients were more likely to be male and older than 60 years (all *p* < 0.001). Non-Hispanic Black patients were more likely to be female and single marital status (all *p* < 0.001). The small intestine (34.6%) neuroendocrine tumor was the most frequent primary site among all gastrointestinal neuroendocrine tumor patients, followed by the rectum (31.2%), colon (12.8%), stomach (11.8%), appendix (8.2%), and esophageal (1.4%) neuroendocrine tumors. Esophageal and colon neuroendocrine tumors presented with high-grade histology (grades 3 and 4) compared to other primary sites (66.5% and 24.9%, respectively). The most frequent primary site of gastrointestinal neuroendocrine tumors in non-Hispanic White patients was the small intestine, whereas for Hispanic and non-Hispanic Black patients, it was the rectum (Figure 1). Compared to others, Hispanic patients had a significantly higher proportion of stomach neuroendocrine tumors (*p* < 0.001), while non-Hispanic Black patients had a higher rate of rectal neuroendocrine tumors (*p* < 0.001) (Table 1). The size of the tumor at presentation of the American Joint Committee on Cancer (AJCC) staging (stage III/IV) was higher in non-Hispanic White patients; they were also more likely to present with regional and metastatic disease (all *p* < 0.001). Non-Hispanic White patients were also more likely to undergo surgery for gastrointestinal tract neuroendocrine tumors (*p* < 0.001) (Table 1). Not surprisingly, radiation therapy and chemotherapy were infrequently used in the treatment of gastrointestinal tract neuroendocrine tumors, as they are not primary treatments for this cancer. However, a statistically significant difference in the receipt of such modalities was noted between non-Hispanic White patients as compared to non-Hispanic Black and Hispanic patients. Patient and tumor characteristics are summarized in Table 1.

### 2.2. Survival Analysis

#### 2.2.1. Overall Survival Analysis (Entire Cohort)

**Figure 2:**
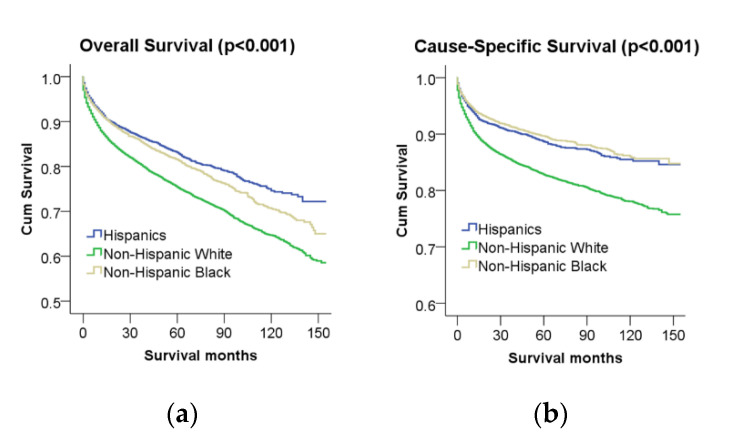
(**a**) Racial disparities in overall survival; (**b**) racial disparities in cause-specific survival.

The 5-year overall survival rate was the highest for appendix and rectum neuroendocrine tumors (both 90%), followed by the small intestine (77%), stomach (68%), colon (58%), and esophageal (8%) neuroendocrine tumors. When different ethnicities/races were compared, Hispanic patients had better overall survival compared to non-Hispanic White and non-Hispanic Black patients (*p* < 0.001). The 5-year overall survival rate was 83% for Hispanic patients, 81% for non-Hispanic Black patients, and 75% for non-Hispanic White patients. The 10-year overall survival rate was 74% for Hispanics patients, 70% for non-Hispanic Black patients, and 64% for non-Hispanic White patients (Table 1, Figure 2a). On multivariable analysis, Hispanic patients were still associated with better overall survival than non-Hispanic White patients (HR: 0.89 (0.81–0.97)) (Table 2). However, there was no significant survival difference between non-Hispanic White and non-Hispanic Black patients.

#### 2.2.2. Cause-Specific Survival Analysis (Entire Cohort)

Cause-specific survival censors out the effect of mortality from other causes than the disease. In the gastrointestinal neuroendocrine tumor, Hispanic and non-Hispanic Black patients had better cause-specific survival than non-Hispanic White patients (*p* < 0.001), and there was no difference between non-Hispanic Black and Hispanic patients. Five-year cause-specific survival was 90% for non-Hispanic Black patients, 88% for Hispanic patients, and 83% for non-Hispanic White patients. The 10-year cause-specific survival rate was 86% for non-Hispanic Black patients, 85% for Hispanic patients, and 78% for non-Hispanic White patients (Figure 2b). After multivariable Cox regression analysis, race/ethnicity was independently associated with cause-specific survival. Non-Hispanic Black patients had better cause-specific survival compared to non-Hispanic White patients (0.89 (0.80–0.98)) (Table 2).

#### 2.2.3. Overall Survival and Cause-Specific Survival for Patients Stratified by Primary Site

**Figure 3:**
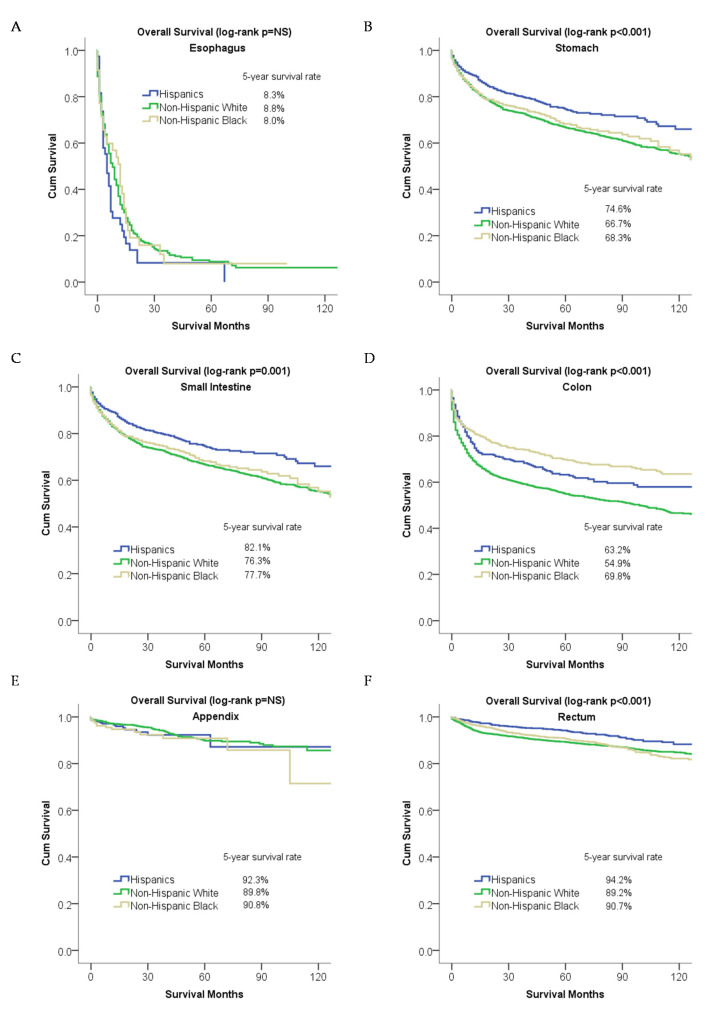
Racial disparities in overall survival for patients stratified by primary site: (**A**) esophagus, (**B**) stomach, (**C**) small intestine, (**D**) colon, (**E**) rectum, and (**F**) appendix.

**Figure 4:**
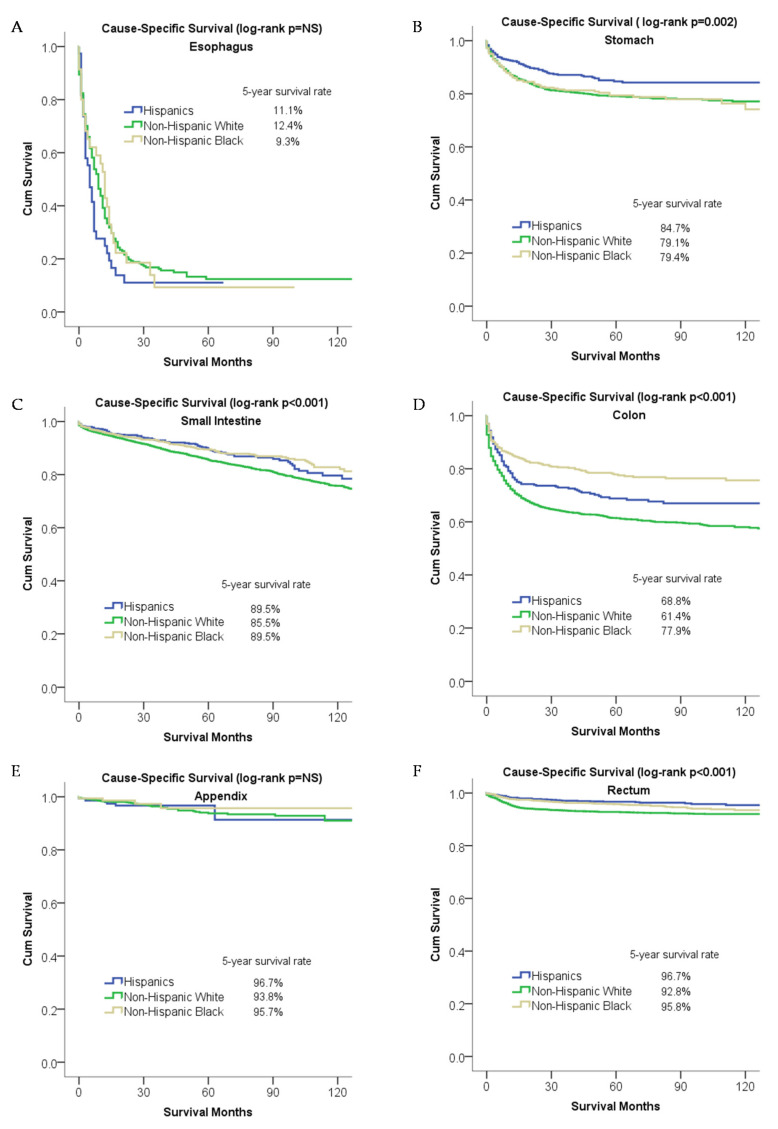
Racial disparities in cause-specific survival for patients stratified by primary site: (**A**) esophagus, (**B**) stomach, (**C**) small intestine, (**D**) colon, (**E**) rectum, and (**F**) appendix.

Hispanic patients had better overall survival in the stomach, small intestine, and rectal neuroendocrine tumors, while non-Hispanic Black patients had better overall survival in colonic neuroendocrine tumors (Figure 3). The Table S1 describes the effect of race/ethnicity on overall survival for each primary anatomical site independent of other variables. In the multivariable analysis, Hispanic patients were associated with better overall survival in the small intestine (0.81 (0.69–0.96)) and rectum (0.79 (0.63–0.99)) as compared to non-Hispanic White patients (Table S1). A similar finding was observed for univariable cause-specific survival analysis (Figure 4). On multivariable analysis, non-Hispanic Black patients were associated with better cause-specific survival in the small intestine as compared to non-Hispanic White patients (0.73 (0.62–0.88)) (Table S1).

## 3. Discussion

This study used the SEER database to examine the largest series of patients with gastrointestinal tract neuroendocrine tumors, comparing their survival outcomes based on race/ethnicity. The current analysis is unique as it includes detailed information about the Hispanic population with gastrointestinal neuroendocrine tumors, as this group had limited information in prior reports. The result indicates that Hispanic patients had better overall survival than non-Hispanic White patients, while non-Hispanic Black patients had better cause-specific survival than non-Hispanic White patients. This difference persisted after adjusting for potential confounding factors. The lower survival in non-Hispanic White patients was observed despite increased use of surgical, radiation, and chemotherapy treatment modalities. The variables that explain these differences include the site of origin, rate of metastases at presentation, stage of the disease, and age of the non-Hispanic White population compared to the rest.

There were significant racial/ethnic differences identified in the pattern of gastrointestinal neuroendocrine tumors in the present study. The Hispanic patients were significantly younger than other ethnicities at the time of the presentation. Non-Hispanic White patients had a higher frequency of small intestine and colon neuroendocrine tumors, while non-Hispanic Black patients had a higher rate of rectal neuroendocrine tumors. Our results corroborate results from older studies that showed the small intestine neuroendocrine tumor had a higher rate of metastasis at presentation, while stomach and rectal neuroendocrine tumors were present with localized disease [17]. Similarly, colon neuroendocrine tumors had higher tumor grade and behaved more aggressively than rectal neuroendocrine tumors. Yao et al., evaluated the patients from the SEER database diagnosed between 1973 and 2004 and found that rectal neuroendocrine tumors were frequent in Black patients [18]. Hauso et al., compared the Norwegian Registry of Cancer and SEER databases for the patients with neuroendocrine tumors reported in 1993–2004 to assess the racial differences. They found that the incidence of the rectal neuroendocrine tumor was found to be three- to six-fold higher in Black patients than Caucasians [19]. The biological or genetic reasons for the racial differences in age of onset and the site of origin of the gastrointestinal neuroendocrine tumor are unknown at this time.

We then performed multivariable survival analysis to identify independent predictors of overall survival and cause-specific survival. Significant factors of survival included age, sex, race, primary tumor site, tumor histology, marital status, tumor size, previous treatments, disease stage, and tumor grade. This confirmed results from a previous analysis that showed worse cause-specific survival in gastrointestinal tract neuroendocrine tumors patients with a lack of insurance, higher tumor grade, greater tumor size, and metastatic disease [17]. With regard to the racial/ethnic differences on overall survival and cause-specific survival associated with gastrointestinal tract neuroendocrine tumors, we found that Hispanics and non-Hispanic Black patients had better survival outcomes than non-Hispanic White patients. The subgroup analysis based on the primary site showed that non-Hispanic Black patients had better cause-specific survival in the small intestine neuroendocrine tumor compared to non-Hispanic White patients. A study from the United States Neuroendocrine Tumor Study Group with a multi-institutional database reported on the effect of racial disparities on clinical outcomes in 1143 patients surgically-resected gastroenteropancreatic neuroendocrine tumors. They compared the non-Hispanic White and non-Hispanic Black patients only and did not include the Hispanic patient population. It showed better disease-free survival in non-Hispanic Black patients despite having a higher lymph node involvement [20]. Another study from Shen et al., used SEER and SEER-Medicare databases to describe racial differences in the incidence and survival of patients with all distant stage neuroendocrine tumors. Compared to our results, the authors identified that Blacks have a higher incidence of neuroendocrine tumors and worse overall survival rate [21]. However, our study as well their data clearly show that neuroendocrine tumor is a heterogeneous disease, and site of origin plays an important role in survival outcomes. The difference was observed in that study, and our results can be attributed to our analyses focusing on non-pancreatic gastrointestinal neuroendocrine tumors, while they focused on all various types of neuroendocrine tumors, which is a heterogeneous population. Our analysis adds to the literature by assessing the impact of confounding factors on race and ethnicity. This survival disparity may be explained by genetic variations among race/ethnicity, but such information is lacking due to the low incidence of this disease [22,23].

This study has several limitations. Underreporting is a potential limitation of retrospective database studies, which might lead to selection bias. There is no patient-level socioeconomic information, and county-level information is not able to provide the socioeconomic status of the individual patients, which may have a significant effect on the patient’s survival. Other clinical details such as comorbidity, family and social histories, concurrent medications, and molecular characteristics are not accessible on the SEER database, which may impact survival outcomes. Information on disease recurrence recurrent information is also unavailable.

## 4. Materials and Methods

The SEER Program of the National Cancer Institute (NCI) is a population-based cancer registry and covers around 35% of the USA population. The SEER program provides the most extensive database, including patient and tumor characteristics, the first course of treatment, and follow-up information to analyze the cancer incidence and outcomes in the United States. This database is updated annually and publicly available with de-identified patient data for researchers; therefore, this study was exempt from the approval of the University of Texas Southwestern Medical Center Institutional Review Board.

Patients with gastrointestinal tract neuroendocrine tumors, diagnosed between 2004 and 2016, were selected from the SEER database in November 2018 submission [24] using SEER*Stat software v8.3.6 [25]. These patients were identified based on the International Classification of Diseases, 10th Revision (ICD-10) primary site code, and ICD for Oncology, 3rd edition (ICD-O-3) histology codes. The primary site codes were as follows: esophagus (C15.0–C15.9), stomach (C16.0–C16.9), small intestine (C17.0–C17.9), colon (C18.0–C18.9, C19.9), rectum (C20.9), and appendix (C18.1) [17,26]. ICD-O-3 codes for histology were as follows: 8013, 8041-8045, 8150-8153, 8155-8156, 8240-8242, 8245-8246, and 8249 [8,11,18,27]. The pancreatic neuroendocrine tumor was not evaluated due to a previous study describing racial disparities in this primary site [16].

**Figure 5:**
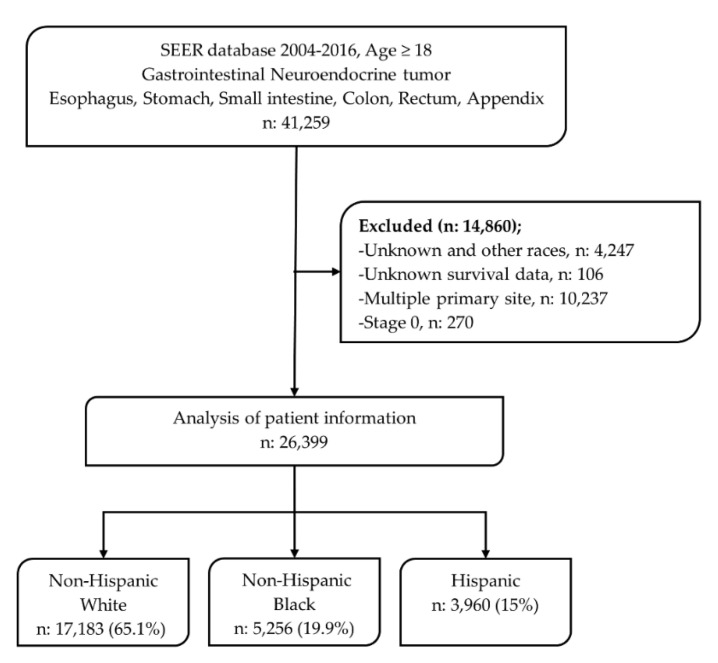
Flow chart to show the selection of patients.

Hispanic, non-Hispanic White, and non-Hispanic Black were the three groups identified for race and ethnicity analyses using SEER race and origin recode [28]. The population of non-Hispanic White, non-Hispanic Black, and Hispanic individuals was significantly larger than the Asian, Native American, and other populations; therefore, we decided to focus on the three groups to maintain robust statistical conclusions. Unknown and other races/ethnicities were excluded [28]. Other variables included were age of diagnosis, sex (male, female), insurance status (insured, uninsured), marital status (married, single, other), primary site, clinical stage (I, II, III, IV), pathological grade, metastatic status (yes, no), tumor size (cutoff size 2 and 4 cm [29]), and treatment history (surgery (yes, no), radiotherapy, and chemotherapy) [7,18,30]. Surgery was included as local or segmental resection based on SEER coding [28]. Receipt of chemotherapy or radiotherapy was coded as “Yes” or “No/Unknown” using SEER recode. The cancer stage was determined by the AJCC staging system 6th or 7th edition. The pathological grade was classified as grade I (well differentiated), II (moderately differentiated), III (poorly differentiated), and IV (undifferentiated or anaplastic) based on the ICD-O-3 grade of differentiation codes. Patients were excluded if they were younger than age 18 years, had stage 0 disease, and multiple primary sites of cancer. Patients missing data on survival were also excluded from the analysis (Figure 5).

The association between demographics and tumor characteristics among various race/ethnicity groups was determined by Chi-square and Fisher’s exact test. Bonferroni correction was used to adjust a significance level for multiple pairwise comparisons. The comparison for overall survival and cause-specific survival was performed by using the Kaplan–Meier method and log-rank test. Overall survival was defined as survival from the date of diagnosis to any cause of death, while cause-specific survival was defined as survival from the date of diagnosis to death related to gastrointestinal tract neuroendocrine tumors [16]. Patients were censored if they died due to other causes or still alive at the last contact date for the cause-specific survival [16]. The 5-year survival rates were compared because the median survival had not been reached at the time of the analysis. Cox proportional hazards model was used for multivariable survival analysis adjusting for age, sex, marital status, insurance, primary site, grade, stage, tumor size, surgery, radiotherapy, and chemotherapy. Missing data for each variable were handled as “unknown” categorical variables and included in multivariable analysis to determine the association between missing data and survival [31,32,33]. SPSS version 25 was used for all statistical analyses, and differences with *p* < 0.05 were considered statistically significant.

## 5. Conclusions

This study suggests that race and ethnicity are important prognostic factors for survival outcomes in gastrointestinal tract neuroendocrine tumors. Hispanic patients had a more favorable overall survival rate than non-Hispanic White patients, whereas non-Hispanic Black patients had better cause-specific survival compared to non-Hispanic White patients. This survival disparity can be attributed to differences in the site of origin, age, and stage at presentation between various races/ethnicities.

## Figures and Tables

**Table 1:** Patient and tumor characteristics by race and ethnicity.

Characteristics	Overall Population (%)	Hispanic (%)	Non-Hispanic White (%)	Non-Hispanic Black (%)	*p*-Value ‡		
					(Hispanic vs NHW) *	(Hispanic vs. NHB) *	(NHW vs. NHB) *
	26,399 (100)	3960 (15.0)	17,183 (65.1)	5256 (19.9)			
Age (median)	57	54	59	56			
Year of Diagnosis					<0.001	<0.001	<0.001
2004–2006	4394 (16.6)	549 (13.9)	3057 (17.8)	788 (15.0)			
2007–2009	5272 (20.0)	719 (18.2)	3470 (20.2)	1083 (20.6)			
2010–2012	6107 (23.1)	898 (22.7)	3932 (22.9)	1277 (24.3)			
2013–2016	10,626 (40.3)	1794 (45.3)	6724 (39.1)	2108 (40.1)			
Sex					<0.001	<0.001	<0.001
Male	12,670 (48)	1840 (46.5)	8600 (50.0)	2230 (42.4)			
Female	13,729 (52)	2120 (53.5)	8583 (50.0)	3026 (57.6)			
Marital Status					<0.001	<0.001	<0.001
Married	14,144 (53.6)	2093 (52.9)	10,035 (58.4)	2016 (38.4)			
Single	4721 (17.9)	797 (20.1)	2488 (14.5)	1436 (27.3)			
Insurance Status					<0.001	NS	<0.001
Yes	19,522 (73.9)	2919 (73.7)	12,772 (74.3)	3831 (72.9)			
No	649 (2.5)	150 (3.8)	289 (1.7)	210 (4.0)			
Primary Site					<0.001	<0.001	<0.001
Esophagus	376 (1.4)	38 (1.0)	303 (1.8)	35 (0.7)			
Stomach	3110 (11.8)	732 (18.5)	1918 (11.2)	460 (8.8)			
Small Intestine	9142 (34.6)	870 (22.0)	6674 (38.8)	1598 (30.4)			
Colon	3386 (12.8)	432 (10.9)	2307 (13.4)	647 (12.3)			
Rectum	8230 (31.2)	1583 (39.9)	4297 (25.0)	2350 (44.7)			
Appendix	2155 (8.2)	305 (7.7)	1684 (9.8)	166 (3.2)			
Tumor Size					<0.001	NS	<0.001
<2 cm	12,121 (45.9)	1848 (46.7)	7858 (45.7)	2415 (45.9)			
2–4 cm	4116 (15.6)	440 (11.1)	3025 (17.6)	651 (12.4)			
>4	2015 (7.6)	249 (6.3)	1464 (8.5)	302 (5.7)			
Metastasis					<0.001	NS	<0.001
Yes	4062 (15.4)	441 (11.1)	3040 (17.7)	581 (11.1)			
Stage					<0.001	<0.001	<0.001
I	6193 (23.5)	1148 (29.0)	3702 (21.5)	1343 (25.6)			
II	1657 (6.3)	211 (5.3)	1136 (6.6)	310 (5.9)			
III	3267 (12.4)	306 (7.7)	2457 (14.3)	504 (9.6)			
IV	3485 (13.2)	394 (9.9)	2601 (15.1)	490 (9.3)			
Grade					<0.001	<0.001	<0.001
I	10,358 (39.2)	1647 (41.6)	6738 (39.2)	1973 (37.5)			
II	2181 (8.3)	291 (7.3)	1557 (9.1)	333 (6.3)			
III	1418 (5.4)	183 (4.6)	1056 (6.1)	179 (3.4)			
IV	622 (2.4)	82 (2.1)	469 (2.7)	71 (1.4)			
Surgery					<0.001	NS	<0.001
Yes	20,570 (77.9)	2990 (75.5)	13,579 (79.0)	4001 (76.1)			
Radiotherapy					<0.001	NS	<0.001
Yes	637 (2.4)	68 (1.7)	486 (2.8)	83 (1.6)			
Chemotherapy					<0.001	NS	<0.001
Yes	2099 (8.0)	257 (6.5)	1525 (8.9)	317 (6.0)			
Overall survival					<0.001	0.014	<0.001
5-year survival rate (%)	78	83	75	81			
10-year survival rate (%)	67	74	64	70			
Cause-specific survival					<0.001	NS	<0.001
5-year survival rate (%)	85	88	83	90			
10-year survival rate (%)	81	85	78	86			

NHW: Non-Hispanic White, NHB: Non-Hispanic Black, NS: not significant. ^‡^ Overall *p*-value was < 0.001 for each characteristic. * *p*-value adjusted with Bonferroni correction method. Grade I: Well differentiated, II: Moderately differentiated, III: Poorly differentiated, and IV: Undifferentiated or anaplastic.

**Table 2:** Multivariable Cox regression analysis for overall survival and cause-specific survival.

Characteristics	OS Multivariable Analysis		CSS Multivariable Analysis	
	HR (95% CI)	*p*-Value	HR (95% CI)	*p*-Value
Race/Ethnicity				
Non-Hispanic White	Ref		Ref	
Non-Hispanic Black	1.03 (0.96–1.11)	NS	0.89 (0.80–0.98)	0.01
Hispanics	0.89 (0.81–0.97)	0.008	0.90 (0.80–1.00)	NS
Age				
18–39	Ref		Ref	
40–59	1.70 (1.43–2.01)	<0.001	1.54 (1.25–1.90)	<0.001
60–79	3.53 (2.97–4.19)	<0.001	2.58 (2.10–3.17)	<0.001
80+	9.22 (7.70–11.03)	<0.001	5.77 (4.62–7.19)	<0.001
Sex				
Female	Ref		Ref	
Male	1.23 (1.16–1.30)	<0.001	1.17 (1.09–1.26)	<0.001
Marital Status				
Married	Ref		Ref	
Single	1.41 (1.30–1.52)	<0.001	1.24 (1.12–1.36)	<0.001
Other/Unknown	1.20 (1.13–1.28)	<0.001	1.10 (1.02–1.19)	0.01
Insurance				
Insured	Ref		Ref	
Uninsured	1.75 (1.48–2.06)	<0.001	1.87 (1.54–2.28)	<0.001
Unknown	1.13 (1.06–1.20)	0.001	1.20 (1.11–1.31)	<0.001
Primary Site				
Appendix	Ref		Ref	
Esophagus	1.34 (1.07–1.68)	0.01	1.37 (1.03–1.81)	0.03
Stomach	1.19 (0.98–1.45)	NS	1.07 (0.82–1.39)	NS
Small Intestine	1.22 (1.009–1.47)	0.04	1.21 (0.94–1.55)	NS
Colon	1.62 (1.34–1.96)	<0.001	1.80 (1.40–2.32)	<0.001
Rectum	0.64 (0.53–0.78)	<0.001	0.65 (0.50–0.85)	0.001
Grade				
I	Ref		Ref	
II	1.24 (1.10–1.40)	0.001	1.59 (1.35–1.85)	<0.001
III	3.76 (3.38–4.18)	<0.001	5.14 (4.50–5.87)	<0.001
IV	4.01 (3.52–4.55)	<0.001	5.37 (4.61–6.25)	<0.001
Unknown	1.48 (1.37–1.60)	<0.001	1.88 (1.68–2.11)	<0.001
Stage				
I	Ref		Ref	
II	1.21 (1.04–1.41)	0.01	1.56 (1.21–2.00)	0.001
III	1.39 (1.21–1.59)	<0.001	2.47 (2.00–3.07)	<0.001
IV	3.74 (3.31–4.22)	<0.001	7.78 (6.36–9.52)	<0.001
Unknown	1.19 (1.06–1.33)	0.004	1.43 (1.17–1.75)	0.001
Tumor Size				
<2 cm	Ref		Ref	
2–4 cm	1.29 (1.19–1.40)	<0.001	2.05 (1.82–2.32)	<0.001
>4 cm	1.56 (1.42–1.71)	<0.001	2.51 (2.21–2.85)	<0.001
Unknown	1.09 (1.01–1.18)	0.02	1.61 (1.43–1.82)	<0.001
Surgery				
Yes	Ref		Ref	
No/Unknown	2.05 (1.91–2.19)	<0.001	2.28 (2.09–2.49)	<0.001
Radiotherapy				
Yes	Ref		Ref	
No/Unknown	0.89 (0.79–0.99)	0.04	0.87 (0.77–0.98)	0.02
Chemotherapy				
Yes	Ref		Ref	
No/Unknown	0.75 (0.69–0.81)	<0.001	0.74 (0.68–0.82)	<0.001

OS: Overall survival, CSS: Cause-specific survival, HR: Hazard ratio, CI: Confidence interval, Ref: Reference, NS: not significant.

## Supplementary Materials

The following are available online at https://www.mdpi.com/2072-6694/12/10/2990/s1, Table S1: Subgroup analysis based on primary site, multivariable Cox regression analysis for overall survival and cause-specific survival.
