# Supplementary material for: Racial/Ethnic Disparities and Survival Characteristics in Non-Pancreatic Gastrointestinal Tract Neuroendocrine Tumors
Source: Cancers (Basel). 2020 Oct 15;12(10):2990. doi: 10.3390/cancers12102990 (PMC7602558; doi:10.3390/cancers12102990)
Supplement: Supplementary file 1 [file cancers-12-02990-s001.pdf]

# Supplementary Materials: Racial/Ethnic Disparities and Survival Characteristics in Non-Pancreatic Gastrointestinal Tract Neuroendocrine Tumors

**Suleyman Yasin Goksu, Muhammet Ozer, Muhammad S. Beg, Nina Niu Sanford, Chul Ahn, Benjamin D. Fangman, Busra B. Goksu, Udit Verma, Aravind Sanjeevaiah , David Hsiehchen, Amy L. Jones, Radhika Kainthla and Syed M. A. Kazmi**

**Table S1.** Subgroup analysis based on primary site, multivariable Cox regression analysis for overall survival and cause-specific survival.

| Characteristics | OS (5-Year Survival Rate) | OS Multivariable Analysis | | CSS (5-Year Survival Rate) | CSS Multivariable Analysis | |
|---|---|---|---|---|---|---|
| | | HR (95% CI) | *p*-Value | | HR (95% CI) | *p*-Value |
| Appendix | | | | | | |
| NHW | 89.8 | Ref | | 93.8 | Ref | |
| NHB | 90.8 | 1.31 (0.72–2.38) | NS | 95.7 | 0.73 (0.26–2.05) | NS |
| Hispanics | 92.3 | 1.72 (0.99–2.97) | NS | 96.7 | 1.66 (0.80–3.47) | NS |
| Esophagus | | | | | | |
| NHW | 8.8 | Ref | | 12.4 | Ref | |
| NHB | 8.0 | 1.34 (0.89–2.02) | NS | 9.3 | 1.43 (0.93–2.20) | NS |
| Hispanics | 8.3 | 1.02 (0.70-1.48) | NS | 11.1 | 1.03 (0.70–1.51) | NS |
| Stomach | | | | | | |
| NHW | 66.7 | Ref | | 79.1 | Ref | |
| NHB | 68.3 | 1.04 (0.86–1.26) | NS | 79.4 | 1.13 (0.88–1.45) | NS |
| Hispanics | 74.6 | 0.86 (0.72–1.03) | NS | 84.7 | 0.84 (0.66–1.08) | NS |
| Small intestine | | | | | | |
| NHW | 76.3 | Ref | | 85.5 | Ref | |
| NHB | 77.7 | 0.97 (0.86–1.09) | NS | 89.5 | 0.73 (0.62–0.88) | 0.001 |
| Hispanics | 82.1 | 0.81 (0.69–0.96) | 0.02 | 89.5 | 0.81 (0.65–1.02) | NS |
| Colon | | | | | | |
| NHW | 54.9 | Ref | | 61.4 | Ref | |
| NHB | 69.8 | 0.96 (0.81–1.13) | NS | 77.9 | 0.86 (0.71–1.05) | NS |
| Hispanics | 63.2 | 0.92 (0.77–1.09) | NS | 68.8 | 0.92 (0.75–1.11) | NS |
| Rectum | | | | | | |
| NHW | 89.2 | Ref | | 92.8 | Ref | |
| NHB | 90.7 | 1.12 (0.95–1.32) | NS | 95.8 | 1.07 (0.83–1.38) | NS |
| Hispanics | 94.2 | 0.79 (0.63–0.99) | 0.04 | 96.7 | 0.89 (0.64–1.24) | NS |

NHW: Non-Hispanic White, NHB: Non-Hispanic Black, OS: Overall survival, CSS: Cause-specific survival, HR: Hazard ratio, CI: Confidence interval, Ref: Reference, N/A: not available, NS: not significant.

© 2020 by the authors. Licensee MDPI, Basel, Switzerland. This article is an open access article distributed under the terms and conditions of the Creative Commons Attribution (CC BY) license (http://creativecommons.org/licenses/by/4.0/).
